# An outbreak of dengue virus (DENV) type 2 Cosmopolitan genotype in Israeli travellers returning from the Seychelles, April 2017

**DOI:** 10.2807/1560-7917.ES.2017.22.26.30563

**Published:** 2017-06-29

**Authors:** Yaniv Lustig, Dana Wolf, Ora Halutz, Eli Schwartz

**Affiliations:** 1 Central Virology Laboratory, Ministry of Health, Chaim Sheba Medical Center, Ramat Gan, Israel; 2 Clinical Virology Unit, Hadassah Hebrew University Medical Center, Jerusalem, Israel; 3 Department of Virology Unit of the Microbiology Laboratory, Dana Dwek Children's Hospital, Tel Aviv Sourasky Medical Center, Tel Aviv, Israel; 4 Sackler Faculty of Medicine, Tel-Aviv University, Tel-Aviv, Israel; 5 Institute of Tropical and Travel Medicine, Sheba Medical Center, Tel Hashomer, Ramat-Gan, Israel

**Keywords:** Dengue type 2, Travelers, The Seychelles, Israel, cosmopolitan lineage

## Abstract

Dengue virus infection was diagnosed in six Israeli travellers returning from the Seychelles in April 2017. Phylogenetic analysis identified identical sequences belonging to the Cosmopolitan genotype of dengue virus type 2 in all samples sequenced, thus providing evidence for a probable dengue type 2 outbreak in the Seychelles. This report further demonstrates the role of travellers as sentinels for arboviral infections, especially in countries with limited diagnostic capabilities.

Because of the high number of mosquito species and especially the abundance of *Aedes albopictus* mosquitoes [1], the Seychelles remain under the threat of outbreaks, particularly arboviruses [2]. However, only limited data are available on the types and molecular characteristics of arboviruses circulating in the region. In this report we investigated an outbreak of dengue virus (DENV) infection in six Israeli travellers returning from the Seychelles, a popular tourist destination located in the Indian ocean east of the East African coast with a population of roughly 90,000 inhabitants. 

## Dengue diagnosis in Israeli travellers

Nine Israeli citizens who had travelled to the Seychelles islands reported a febrile illness which started 1–2 days after returning to Israel. They were part of a group of 32 travellers visiting a different island every day between 13 and 22 April 2017. The islands visited, in consecutive order, were: Mahe, Curieuse, Aride, Praslin, La Digue and Moyenne. 

In six of the febrile travellers, dengue fever was confirmed, in four of them by both quantitative (q) RT-PCR [3] or PCR [4] and DENV IgM and IgG antibody capture ELISA (Panbio, Brisbane, Australia), in one only by qRT-PCR, and in one only by positive dengue virus NS1 antigen (Panbio, Brisbane, Australia) and IgM and IgG serology (Table). Since DENV RNA in serum can only be detected for a short time after symptom onset [5-8], DENV RNA in urine and whole blood was tested for samples obtained more than 10 days post symptom onset (Table). qRT-PCR of DENV-1–4 [3] or PCR [4] demonstrated that all five PCR-positve cases had DENV type 2. The remaining three febrile patients did not present to our clinics and therefore were not tested for dengue. 

**Table:** Clinical and diagnostic data of dengue virus-infected Israeli travellers, Seychelles, April 2017 (n = 6)

Patient	Sex/age group	Main symptoms (description)	RT-PCR result (Ct), sample type	Serum sample			
				Time from onset (days)	NS1 early antigen	IgM	IgG
1	F/50–59	Fever + Rash	Pos (26.7), urine	14	Neg	Pos	Pos
2	M/70–79	Fever + Pruritus	Pos (26), urine	16	Neg	Pos	Pos
3	M/60–69	Fever	Pos (31), urine	15	Neg	Pos	Pos
4	F/70–79	Fever + Rash	Neg, serum	10	Pos	Pos	Pos
5	F/70–79	Fever + Rash	Pos (25.6), serum	6	ND	ND	ND
6	M/80–89	Fever + Rash	Pos^a^, whole blood	15	Neg	Pos	Pos

F: female; M: male; ND: not done; Neg: negative; Pos: positive.

^a^ Only PCR was performed and therefore no Ct value is available.

Three additional members of this group of travellers suffered from upper respiratory symptoms (one of them was only febrile). They were examined in our clinic, tested for DENV infection (by qRT-PCR, NS1 and serology) and found to be negative. We were not able to test the remaining asymptomatic travellers. 

## Genetic relationship

From the four RNA samples positive for DENV in qRT-PCR, 2,450 nt spanning the capsid, pre-membrane (prM), membrane (M) and envelope (E) genes were amplified [4]. The raw sequence data were analysed and trimmed to generate a 1,429-nt consensus sequence of the E gene, using Sequencher 5.4 (GeneCodes, Ann Arbor, Michigan), and the DENV E gene sequences were aligned with 23 DENV type 2 E gene sequences obtained from the GenBank database. The accession numbers for reference sequences are specified in the Figure. Phylogenetic analysis was conducted using a neighbour-joining algorithm in MEGA, version 6 [9], with 1,000 replicates for bootstrap testing. A neighbour-joining phylogenetic tree based on the aligned sequence data showed that all four DENV 2 sequences from Israeli travellers were identical and clustered with the Cosmopolitan genotype. Most importantly, strains belonging to lineage I of the Cosmopolitan genotype from China, India and Singapore were found to cluster with the strains of the present study (Figure).

**Figure:**
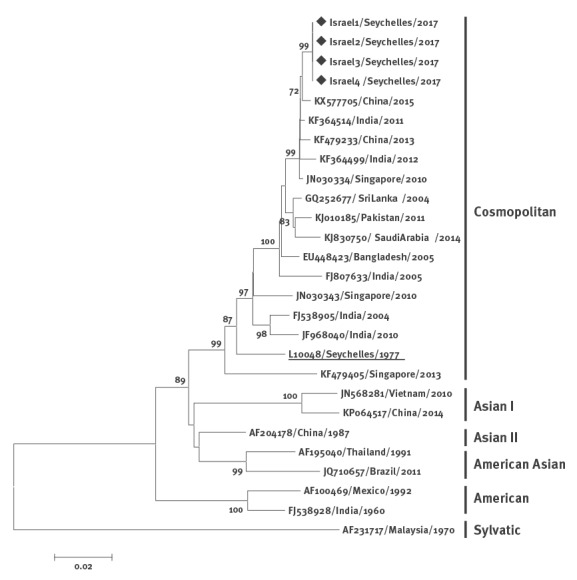
Phylogenetic analysis of the envelope gene sequence of DENV type 2 from Israeli travellers, Seychelles, April 2017 (n = 4)

The analysis was conducted on a nucleotide sequence of the genes encoding the E protein (1,429 bp), using the neighbour-joining method implemented in MEGA 6.0 software. The robustness of branching pattern was tested by 1,000 bootstrap replications. The percentage of successful bootstrap replicates is indicated at nodes, showing only values of > 70%. The bar denotes 0.02 nucleotide substitutions per site. DENV strains sequenced in this study are marked by black diamonds. Reference strains are indicated by accession number and place and year of isolation. The DENV type 2 strain isolated in 1977 in the Seychelles is underlined.

## Discussion

DENV is currently a leading cause of illness and death in the tropics and subtropics with as many as 400 million people infected yearly [10]. However, because it occurs in many areas with limited diagnostic resources and co-circulates with other arboviruses with similar clinical manifestations, travellers are often the most sensitive sentinels for arboviral outbreaks. Here we report a dengue outbreak in the Seychelles that was revealed by six Israeli travellers diagnosed with acute DENV infection, demonstrating the power of such passive arbovirus surveillance. 

Mahe is the largest and most populated island where most of the previous outbreaks in the Seychelles have been reported [11]. Assuming that DENV exposure happened there would result in an incubation time of 10–12 days, which is longer than what is usual for dengue (4–7 days) [12]. Therefore, and because all travellers had disease onset almost on the same day, a simultaneous exposure on one of the other islands is more plausible. The immediate result of this study was an alert through ProMED [13] assuming that this would also alert the Seychelles public health authorities of the possibility of an outbreak on one of the smaller islands.

Although DENV RNA was identified in five patients, NS1 antigen was not detected. This is not surprising, since all NS1-negative samples had been obtained more than 12 days after symptom onset and it has been demonstrated previously that samples from dengue patients do not test positive for NS1 later than 12 days post symptom onset [14]. While detection of DENV RNA in serum is also limited to a short time (< 1 week) after symptom onset, RNA in urine and whole blood can be detected for a longer period, similarly to other flaviviruses such as West Nile and Zika virus [5-8]. Indeed, in our patients, urine and whole blood samples obtained more than 14 days post symptom onset were positive for DENV RNA.

During the past 5 years, two outbreaks of dengue or dengue-like symptoms have been documented in the Seychelles and both DENV types 1 and 2 have been identified [11]. However, DENV was isolated only once in 1977 (GenBank accession number: L10048) and found to be type 2 [15]. Phylogenetic analysis performed in 1999 identified that this 1977 DENV strain was most similar to dengue 2 strains isolated in Delhi, India in 1996 [16]. Our data demonstrate that the 1977 isolate similarly to the 2017 isolates, belonged to the Cosmopolitan genotype. It is tempting to speculate that the Cosmopolitan genotype has continued to circulate in the Seychelles from the 1970s until now. However, because the phylogeny differed considerably between the current and former DENV-2 strains from the Seychelles and similar cosmopolitan strains were identified in China in 2013 and 2015 and in India in 2011–12, we hypothesise that the DENV responsible for the current outbreak was imported from a country endemic for Cosmopolitan DENV-2.

## Conclusion

This study identified a DENV outbreak in the Seychelles. The diagnosis of six individuals with DENV infection among 32 Israeli travellers suggests that there is considerable circulation of DENV in mosquitoes in the Seychelles. Future monitoring of DENV circulation in mosquitoes in this area should reveal the extent of penetration of DENV and other arboviruses in the Seychelles and will contribute considerably to the epidemiological characterisation of these zoonotic viruses.
